# Isolation, screening, and molecular identification of endopytic fungus producing cellulose and cyanide degrading enzyme its application for waste cassava

**DOI:** 10.5455/javar.2025.l884

**Published:** 2025-03-25

**Authors:** Yetti Marlida, Husmaini Husmaini, Ahadiyah Yuniza, Lili Anggraini, Wulansih Dwi Astuti, Ridho Kurniawan Rusli, Hera Dwi Triani, Gusri Yanti

**Affiliations:** 1 Department of Animal Nutrition, Faculty of Animal Science, Universitas Andalas, Padang, Indonesia; 2 Department of Animal Production, Faculty of Animal Science, Universitas Andalas, Padang, Indonesia; 3 Research Center for Animal Husbandry, National Research and Innovation Agency (BRIN), Cibinong, Indonesia; 4 Research Center for Applied Zoology, National Research and Innovation Agency (BRIN), Cibinong, Indonesia; 5 Department of Agricultural Extension, Faculty of Social, Science and Education, Universitas Prima Nusantara, Bukittinggi, Indonesia

**Keywords:** Cellulose, endophytic fungus, identification, isolation, screening

## Abstract

**Objective:**

This research aims to isolate, screen, and identify some candidates for endophytic fungus-producing cellulase and cyanidase.

**Materials and Methods:**

Fungi were isolated from cassava leaves that had undergone surface sterilization. The fungal isolates were qualitatively tested for their ability to produce cellulase and cyanidase enzymes by adding carboxy methyl cellulose (CMC) and KCN to the media. Enzyme production was indicated by the formation of clear zones around the growing colonies. Isolates that tested positive for cellulase and cyanidase production underwent further quantitative screening to measure enzyme activity using a spectrophotometer at wavelengths of 540 nm and 400 nm, respectively. The isolates showing the highest cellulase and cyanidase activity were identified through 18S rRNA analysis using the Sanger DNA sequencing method.

**Results:**

The research obtained six pure isolates of endophytic fungus, namely Y1; Y2; Y3; Y4; Y5; and Y6. Four isolates had the ability to degrade CMC with a clear zone between 0.1 until 0.5 mm, and three isolates had the ability for KCN degrade. The highest activity for cellulase and cyanidase degrading enzymes was produced by isolate Y2. After molecular identification using 18S rRNA, isolate Y2 had 98.82% similarity to *Phomopsis* sp. 32PG/F.

**Conclusion:**

Six isolates of endophytic fungi were obtained, Y1; Y2; Y3; Y4; Y5; and Y6. Four isolate the ability of to degrade CMC and three isolate the ability for KCN degrade. Isolate Y2 is the isolate with the best activity for cellulase and cyanidase degrading enzymes, namely 2.99 U/ml and 2.19 U/ml. After molecular identification using 18S rRNA, isolate Y2 had 98.82% similarity to *Phomopsis* sp. 32PG/F.

## Introduction

West Sumatra is a province with an abundance of natural resources, such as cassava plants. Statistical data for 2021 reports total cassava production in West Sumatra which is 153. 412.02 tons, where the highest production is in 50 Kota District, followed by Agam District, where 5%–15% of cassava tubers are skin. The main problem using cassava waste as feed is the high crude fiber content (21% DM) and cyanide acid hydrocyanic acid (HCN) as well as low protein content (4.8% DM); therefore, it cannot be given to livestock in large quantities without the application of advanced biotechnology, such as fermentation and enzymatic processes. Drying is the most common method used to reduce the cyanide content of cassava peel. Williams et al. [1] in their study explained that drying cassava peel at room temperature results in the highest reduction of cyanide levels compared to boiling.

Physical and chemical treatments have been unable to reduce cyanide and improve its nutrients from cassava waste (skin and leaves) to be used as ingredients in rations energy source that partially replaces corn. A group of researchers reported that fermenting cassava skin using bacteria, mold, and yeast can reduce cyanide/HCN and fiber crude and increase protein and amino acid content, thus the beneficial value of waste. This increases and can be used as more feed ingredients in the composition ration. Gunun et al. [2] reported an increase in cassava peel protein using yeast (*Saccharomyces cerevisiae*), providing increased protein results from 2.1% to 13.7%–13.8% for 14 days. In addition, silaged cassava leaves can also reduce cyanide content from 62% to 80.6% and can be stored for up to 90 days [3]. Li et al. [4] reported that Paenibacillus and Bacillus sp. were found in cassava foliage silage.

The potential of cassava leaves as a source of animal feed is also no less important because Cassava leaves are a very rich source of crude protein, dietary fiber, polyunsaturated fatty acid, bioactive compounds, and vitamins [5,6]. Production of cassava leaves is estimated at 10 tons of dry leaves per hectare, which has a high yield the same as tubers. Cassava leaves contain approximately protein ranging from 11.8% to 38.1% [7] and anti-nutritional compounds, cyanogenic potential, phytate, and tannin, ranging from 1.4 to 1.9 gm/100 gm: 0.6% to 3.1% and 22.2 to 28.7 mg/100 gm, respectively [8]. Conventional methods have been proven not to be effective in reducing the cyanide content in cassava leaves to safe limits while causing significant loss of protein and essential nutrients, which are very desired from cassava leaves. Therefore, the method that produces edible cassava leaves with low cyanide levels while maintaining maximum nutritional content has not yet been exploited in depth.

One of the important criteria for solid media fermentation is using cassava waste is the selection of appropriate microorganisms, for this reason, this paper is looking for the right mold that comes from the cassava plant itself, namely endophytic fungi. Endophytic fungi are a group of fascinating host-associated fungal communities that colonize the intercellular or intracellular spaces of host tissues, providing beneficial effects to their hosts while gaining advantages [9]. Imaningsih et al. [10] isolated endophytic fungi originating from plants were able to grow in acidic conditions. In this research, isolation will be carried out characterization and optimization of endophytic fungi which are able to reduce crude fiber and cyanide a mixture of cassava skin and leaves and increase nutritional value such as protein and acid amnio.

## Materials and Methods

### Ethical approval

Ethical approval was not required because this study did not use any live animals.

### Isolation of fungal endophytes

Cassava leaves and stems are washed with running water. Leaf segments are cut evenly with a sterilized using an scalpel, starting from the center of the healthy leaf to the midrib. The cut segments were surface sterilized by immersing them in the following series of solutions: sterile distilled water for 60 sec, 70% ethanol for 60 sec, 2.5% sodium hypochlorite for 4 min, 70% ethanol for 30 sec, and final rinsing in a sterile distilled water three times. Sterilized cassava leaves were cut into five segments (5 mm), and 20 leaf segments per plant were placed on the surface of PDA agar supplemented with 0.05 gm of streptomycin sulfate per 100 ml of medium to inhibit bacterial growth. The plates were incubated at 28°C and monitored daily for mold growth. Single isolates growing from tissue were re-inoculated on PDA agar media (modified method AlKahtani et al.) [11].

### Screening of isolates for their cellulolytic and cyanidase activity

All endophytic fungal isolates were subjected to qualitative tests of extracellular cellulase and cyanidase. Screening of cellulolytic fungi was based on Rahardiyan and Moko [12] with a few modifications. The media composition included 0.02 gm of MgSO_4_.7H_2_O; 0.075 gm of KNO_3_; 1 gm of carboxy methyl cellulose (CMC), 0.05 K_2_HPO_4_; 0.002 gm of FeSO_4_; 0.004 gm of CaCl_2_; 0.2 gm of bacto agar and 0.1 gm of glucose per 100 ml of distilled water. The cultures were at 37°C for 24 h. After invubation, 0.1% Congo Red solution (w/v) was poured over the culture and left for 15 min. The solution was discarded and rinsed with 0.2 M NaCl for 15 min. Fungal isolates that capable of decomposing cellulose werwindicated by forming a clear zone around the colony. The clear zone, observed as hydrolysis halo surrounding the colonies, indicated lignocellulolytic activity [13,14]. The selection of cyanide-producing fungi began by subculturing fungal colonies on bacto-agar containing 25 mg/l KCN. Enrichment media were with the following composition: NaHPO_4_—4.0 gm, Na_2_SO_4_—2.13 gm, K_2_HPO_4_—3.1 gm, MgCl_2_.6H_2_O—200 mg, —FeCl_3_.6H_2_O—2.0 mg, and CaCl_2_—1.0 mg in 1 l of distilled water and the pH was adjusted to 7.2. Observe were conducted over 3–5 days. Fungal isolates capable ofdecomposing cyanide were indicated by formation of a clear zone around the colonies.

### Production of enzyme and extraction

Production of cellulase enzymes was conducted submerged fermentation in a 250 ml Erlenmeyer flask containing 100 ml of fermentation media. The composition of the fermentation media contained per 100 ml of distilled water: 0.02 gm; MgSO_4_.7H_2_O; 0.075 gm KNO_3_; 0.05 gm K_2_HPO_4_; 0.002 gm FeSO_4_; 0.004 gm of CaCl_2_ and 0.1 gm of glucose and 1 gm of CMC. The cultures were incubated at 37°C for 7 days. After incubation, the samples were centrifuge at 4°C for 20 min at 10,000 rpm. The enzyme extract was collected and prepared for use. Production of cyanidase enzymes was carried out using p-NPG (p-Nitrophenyl Glucopyranoside) 0.1% (b/v) for cyanidase. The pH of the medium was adjusted to 5. The medium was sterilized by autoclaving at 121°C for 15 min. Each flask was inoculated with a loopful of the prepared inoculum. The culture were incubated on a rotary shaker at 120 rpm and 30°C for 7–8 days. Enzyme extraction: after the fermentation period, the culture broth from submerged fermentation was centrifuged at 6,000 rpm for 15 min. The resulting supernatant was collected and used as the crude enzyme extract.

### Assay for cellulase activity

An aliquot of 0.5 ml of cell-free supernatant was transferred to a clean reaction tube and 0.5 ml of 1% CMC (w/v) in 0.05 M Sodium citrate buffer (pH 4.8) was added. The tube was incubated in a water bath at 55°C for 15 min. After incubation, 3 ml of DNSA reagent was added to stop the reaction. The reactants in the test tube were incubated in a boiling water bath for 10 min. After incubation, the liberated sugars were determined by measuring the absorbance at 540 nm in a colorimeter. Enzyme activity is determined in international units which is defined as the amount of enzyme that produces 1 μ mole of glucose per minute [15].

### Assay for glucosidase activity

Enzyme extract, pH 5.0 citrate buffer, and substrate were preincubated for at 50°C 10 min. Subsequently, 0.5 ml of enzyme extract was mixed with 0.5 ml of citrate buffer and 0.5 ml of substrate. The mixture was then incubated at 50°C for 60 min. To stop the reaction, 1 ml of 1M Na2CO_3_ solution was added, and the mixture was vortexed thoroughly. The absorbance was measured using a spectrophotometer at a wavelength of 400 nm. A control was prepared with the same composition, but the enzyme was added only after the 1 ml of 1M Na₂CO₃ solution. For the blank, 1 ml of distilled water, 0.5 ml of buffer solution and 1 ml of Na_2_CO_3_ were used. A standard curve was generated using p-nitrophenol as the standard solution.

### Molecular identification of endophytic fungi

Identification was conducted using 18S rRNA. DNA extraction was performed using the KIT Quick-DNA Magbead Plus Kit (Zymo Research, D4082). The quality of the extracted DNA was assessed using nanodrop spectrophotometry. The DNA extraction results were amplified using kit MyTaq HS Red Mix, 2X (Bioline, BIO-25048). One µl of amplification results were analyzed using 1% agarose gel electrophoresis in Tris-borate-EDTA buffer to visualize the PCR results. Amplified ITS gene products were subjected to electrophoresis. Sequencing was performed using the Sanger DNA Sequencing method with Capillary Electrophoresis.

## Results

### Isolation and screening of endophytic fungi

Endophytic fungi produce enzymes and various bioactive secondary metabolites with significant industrial value. They have the capability to degrade or sequester inorganic and organic molecules, including small molecules and macromolecules such as toxins, pollutants, and heavy metals. Additionally, these fungi exhibit the ability to perform highly selective catalytic conversion of high-value compounds in an environmentally friendly manner. This characteristic makes them valuable for the production/innovation of bioactive molecules, food and nutrition, agriculture, and the environment. Endophytic microbes are derived from within plants, where they play a beneficial role by producing primary and secondary metabolites that help plants defend against diseases [16].

**Figure 1.**
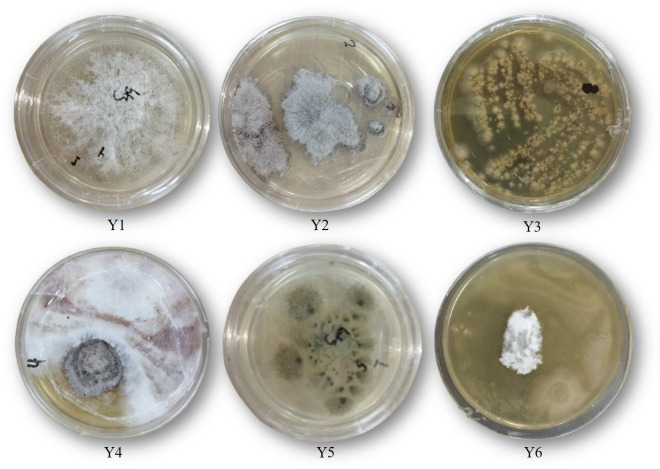
Endophytic fungus isolates isolated from cassava plants. Six isolates were successfully isolated: Y1, Y2, Y3, Y4, Y5, and Y6.

**Figure 2.**
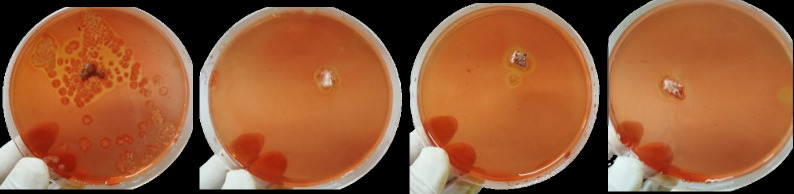
Qualitative testing of endophytic fungi producing cellulase. Four out of the six isolates produced clear zones on CMC-containing media.

**Figure 3.**
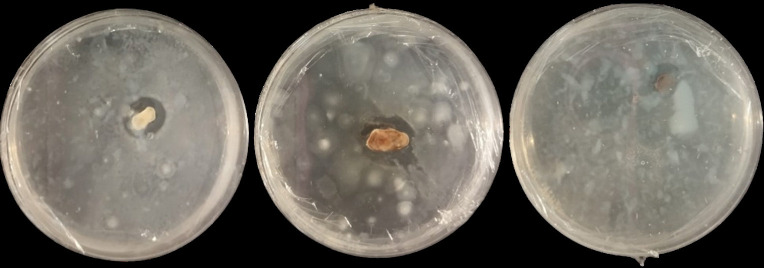
Qualitative testing of cyanide degrading molds. Three out of the six isolates produced clear zones on KCN-containing media.

In this research, endophytic fungi were isolated from the leaves of cassava plants to explore their potential for producing cellulase and cyanidase. The culture isolation and purification process resulted in six isolates of endophytic fungi obtained from cassava leaves (Fig. 1). The enzymatic potential of these six isolates was first assessed for their ability to degrade cellulose, followed by an evaluation of their cyanide-degrading capability (Figs. 2 and 3).

### Capability of endophytic fungi on cellulase and cyanidase degrading enzymes

**Figure 4.**
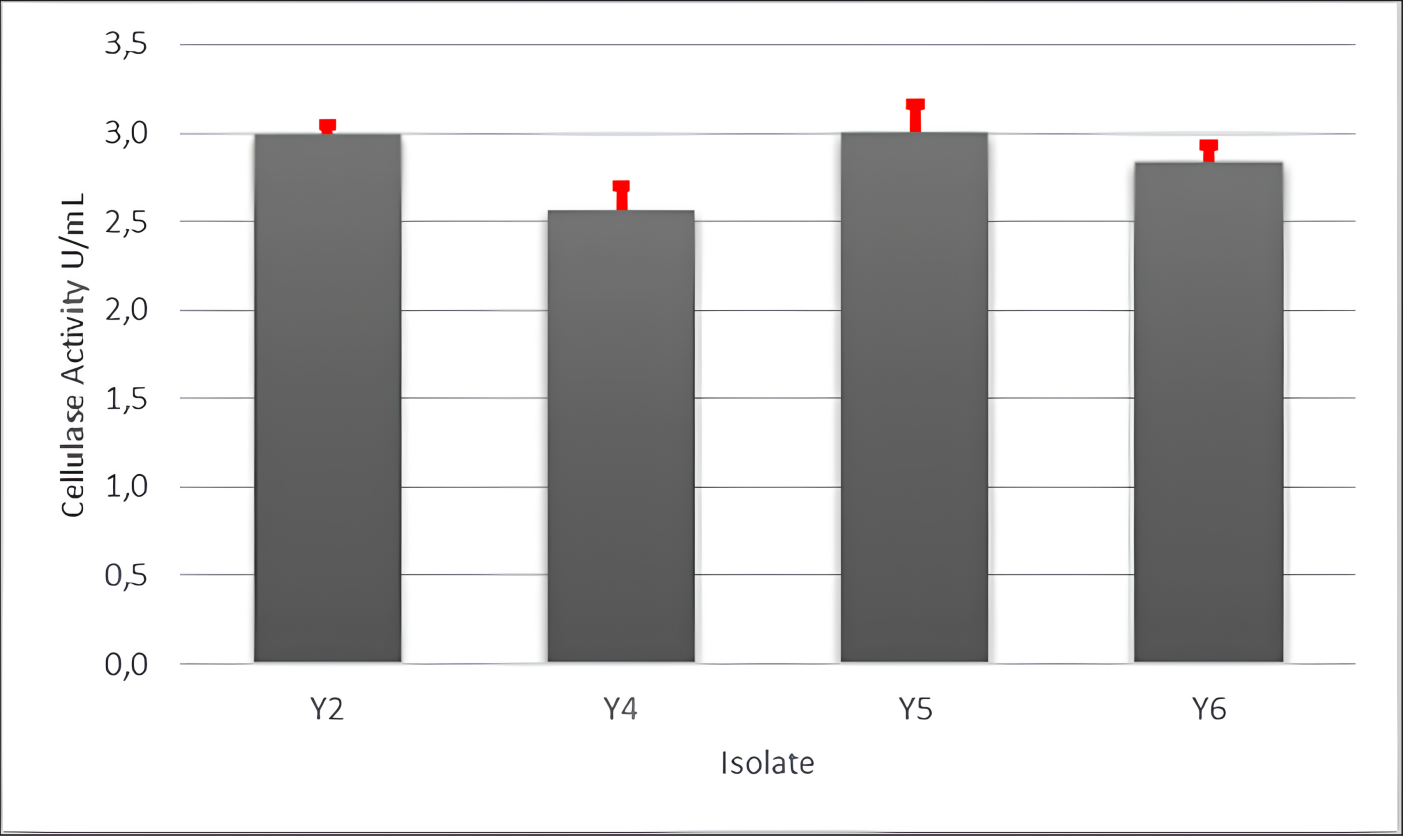
Cellulase enzyme activity of endophytic fungus isolates isolated from cassava plants.

**Figure 5.**
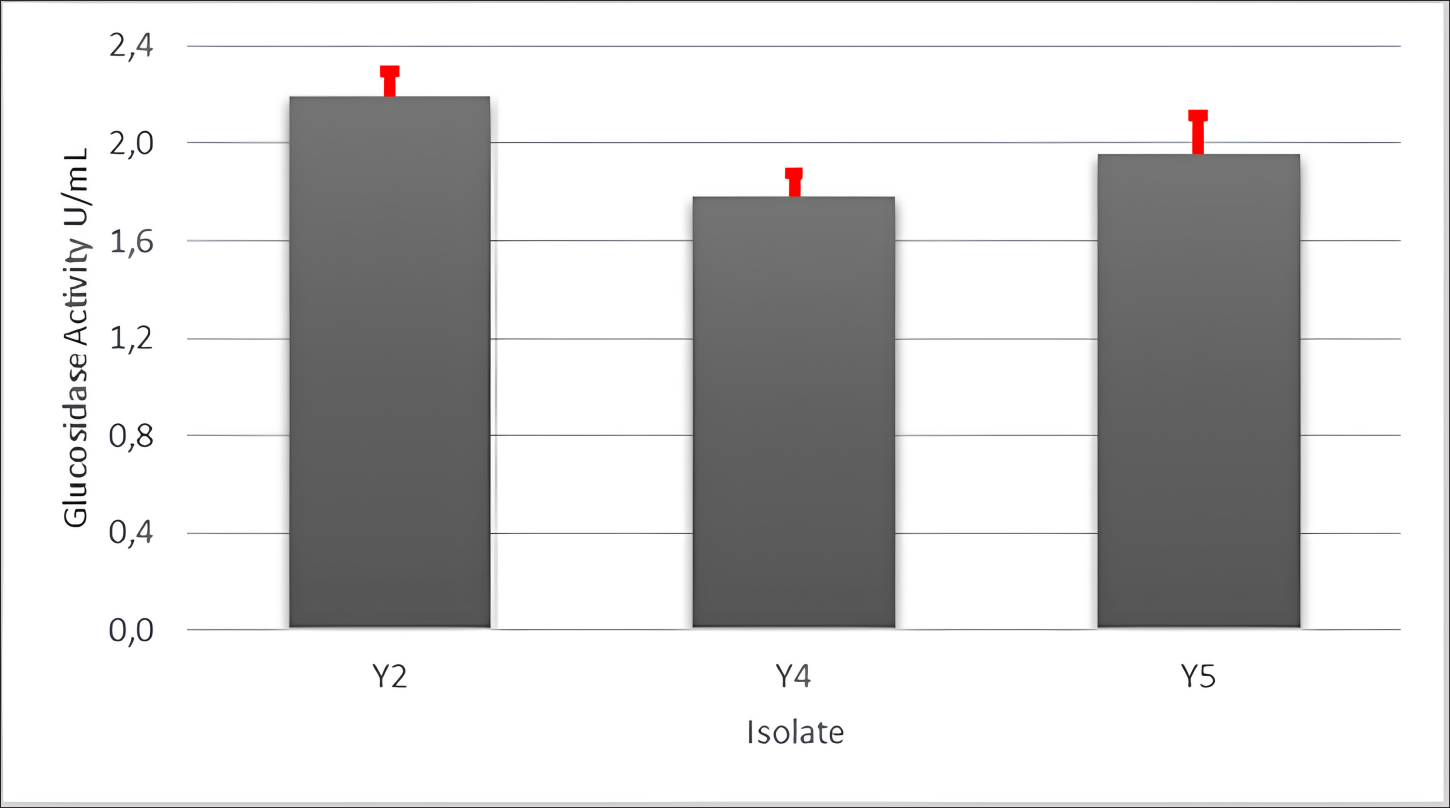
Activity of glucosidase enzyme of endophytic fungus isolates isolated from cassava plants.

After qualitative screening of six endophytic fungal culture isolates, only four isolates demonstrated the ability to degrade CMC in the medium. To assess cellulase activity, these four isolates were inoculated on the cellulase production medium, with CMC as the inducer. The activity of cellulose results are shown in Figure 4. The cyanidase-degrading activity (Fig. 5) was evaluated for the same four isolates capable of producing cellulase. Among them, only three isolates exhibited the ability to degrade KCN, resulting in three isolates capable of producing both cellulase and cyanidase-degrading enzymes. Figures 4 and 5 highlight the selection of isolates with a high capacity to produce both enzymes. Isolate Y2 shows the highest ability to produce both types of enzymes. In Figures 4 and 5, it can be seen that isolate Y2 shows the highest ability to produce cellulase and cyanidase enzymes. Based on these findings, isolate Y2 is recommended for further molecular identification.

### Identification of selected endophytic fungi by molecular

**Figure 6.**
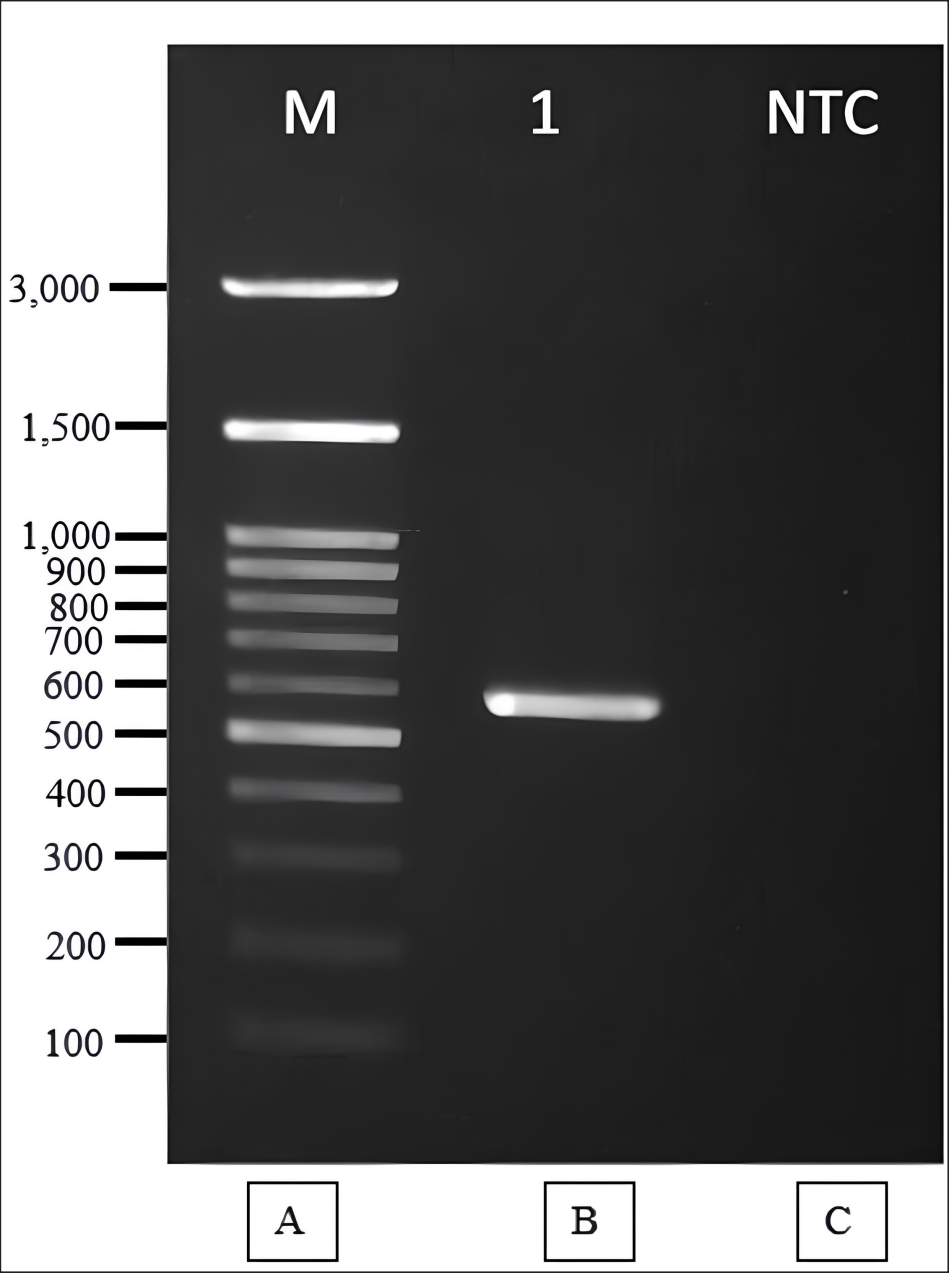
Amplification of 18S rRNA Gene of endophytic fungal isolates. A. DNA Ladder 100bp B. PCR Product C. Negative control of amplification.

**Figure 7.**
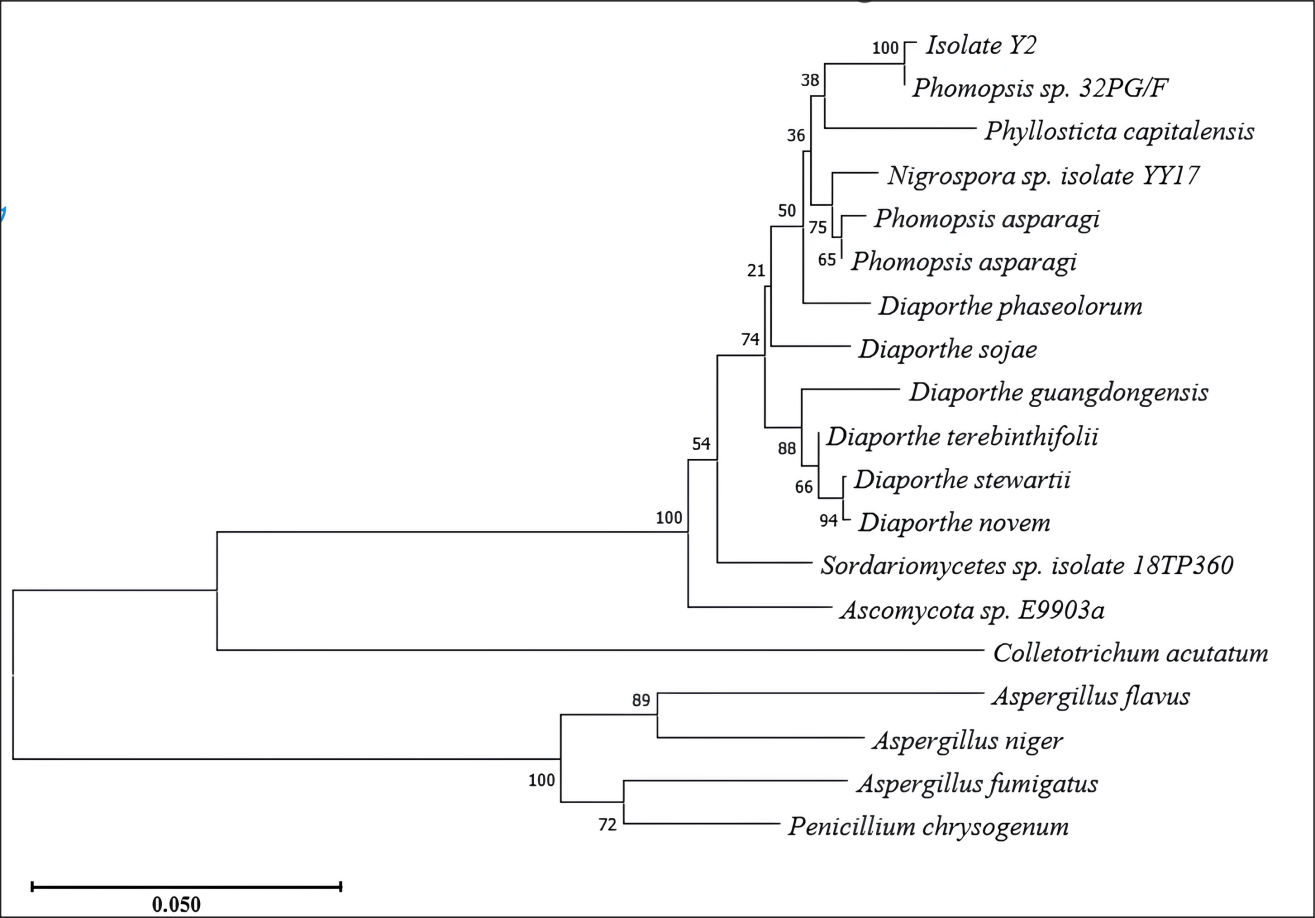
Phylogenetic tree of Y2 isolate.

Identification of isolate Y2 was conducted based on 18S rRNA gene sequence analysis. The process involved DNA isolation, amplification, sequencing, sequence alignment analysis, and phylogenetic analysis. The phylogenetic relationships are depicted in Figure 7, while the amplification results are presented in Figure 6. Phylogenetic analysis revealed that isolate Y2 belongs to *Phomopsis* sp. 32PG/F.

## Discussion

This research successfully isolated six endophytic fungal isolates from cassava leaves (Fig. 1) using the method described by Alkahtani et al. [11]. Endophytes have been isolated from various plants, ranging from mosses to woody trees such as chili pepper and sungkai [10,17]. All plant organs can serve as potential niches for endophytic microorganisms, with roots typically having the highest endophyte density [18]. However, unlike cassava plants, attempted isolation from stems, twigs, and leaves, but the leaves found the endophytic fungi. Another important factor to consider when selecting plants is their health. Collecting samples from healthy plants without visible symptoms minimizes likelihood of isolationg pathogens along with endophytes [19]. Endophytic fungi play a crucial role in protecting plants from environmental stress, enhancing plant growth, safeguarding plants from pathogenic microorganisms, maintaining plant health through the production of bioactive compounds.

The qualitative screening of endophytic fungi for producing cellulase and cyanidase enzymes was conducted using selective media. Agar media containing CMC was used for detecting cellulase enzyme activity, and while KCN-containing media was used for cyanidase enzyme activity. Among the six isolates successfully obtained, four exhibited cellulase enzyme activity, and only three demonstrated simultaneous cyanidase and cellulase enzyme activity.

Cellulose in cassava is one of the main components of plant cell walls and constitutes a type of fiber that cannot be directly digested by livestock, particularly poultry. The degradation of polysaccharides in cassava into glucose can be facilitated by the cellulase, enabling the fiber in cassava to be digestible for poultry. Qualitative testing for cellulose enzymes can be conducted using CMC substrates. In this study, found four isolates (Y2, Y4, Y5, and Y6) were successfully isolated and demonstrated the ability to grow on media containing CMC. (Fig. 2).

Cellulase enzyme activity in hydrolyzing CMC was observed through congo red staining, as indicated by the presence of a clear zone around the growing fungal colonies. CMC is used in cellulase enzyme activity testing because CMC its carbon skeleton is similar to that of cellulose, allowing cellulase enzyme to hydrolyze it effectively [20]. CMC degradation involves a consortium of three enzymes: endo-β-(1,4)-glucanase, exo-(β-1,4)-glucanase, and β-(1,4)-glucosidase. These enzymes collectively reduce the length of the CMC chain, leading to a decrease in fluid viscosity [21,22]. Endo-glucanase hydrolyzes the polymer skeleton, releasing cellobiose, cellotriose, and other oligomers. Exo-glucanases further hydrolyze the ends of these oligomeric polymer chains, resulting in the formation and release of cellobiose, which is subsequently hydrolyzed by β-1,4-glucosidase to form glucose [23].

Naher et al. [24] successfully isolated cellulase-producing fungi from soil samples and weed plants, identifying *Trichoderma reesei *UMK01 and *Aspergillus awamori *UMK02. Similarly, Hawar [25] isolated five types of endophytic fungi from *Ziziphus spina *leaves that exhibited cellulase activity using CMC test media. These fungi were identified as *Aspergillus niger, A. fumigatus, Cladosporium *sp., *Rhizopus* sp., and *Mucor* sp.

Three endophytic fungus isolates from cassava plants, identified as Y2, Y4, and Y5 (Fig. 3), demonstrated cyanidase enzyme activity based on KCN degradation. Cassava plants contain cyanogenic glycoside compounds, especially linamarin and lotastralin. These compounds produce cyanide upon decomposed. Cyanide is a toxic compound that can disrupt cellular function by inhibiting cytochrome c oxidase, a key enzyme in the electron transport chain in mitochondria. The discovery of these cyanide-degrading fungi offers potential solution for processing cassava waste as feed ingredients.

Cyanide biodegradation by microorganisms occurs through four pathways: hydrolysis, oxidation, reduction, and substitution/transfer. Each organism may utilize one or more pathway to degrading cyanide [26,27]. I the hydrolysis, oxidation, and reduction pathway, enzymes catalyze and convert cyanide into the simpler organic and inorganic compounds, such as ammonia ion, methane, CO_2_, formic acid, or carboxylic acid. Through substitution and biosynthesis, microorganisms assimilate cyanide as a source of carbon and nitrogen [28]. Several microbial strains capable to degrade cyanide, such as *Trichoderma harzianum, Fusarium oxysporum, Alternaria* sp., *Chaetomium globosum* [29], *Aspergillus niger* [30].

Cellulase activity was assessed on four fungal isolates capable of degrading CMC. CMC degradation is carried out by the enzyme endo-(1,4)-β-D-glucanase. The breakdown of cellulose involves a synergy action of cellulase enzymes: (1) endo-(1,4)-β-D-glucanase (endoglucanases), (2) exo-(1,4)-β-D-glucanase (exoglucanases), and (3) β -glucosidase [31,32].

Cellulase enzyme activity was tested on endophytic fungus isolates capable of producing cellulase in solid media, specifically isolates Y2, Y4, Y5, and Y6 (Fig. 2). Meanwhile, glucosidase enzyme activity was evaluated in positive isolates producing cyanidase enzymes, namely Y2, Y4, and Y5 (Fig. 3). Among these, isolate Y5 exhibited the best cellulase enzyme activity at 3.01 U/ml, while isolate Y2 demonstrated the highestglucosidase enzyme activity at 2.19 U/ml (Fig. 5).

Testing the activity of fungal cellulase enzymes using CMC as a substrate. Fungal cellulase enzymes consist of two main domains; the catalytic domain (CD) and the carbohydrate-binding domain (CBD). These domains play distinct roles, the CBD aiding the enzyme in the substrate-binding process, while the CD is responsible for the hydrolysis reaction [33]. Cellulase enzyme complex belong to the glycoside hydrolase (GH) family of enzymes, whose primary function is the hydrolysis polysaccharide. The enzyme complex acts on different parts of cellulose, and their synergistic action converts cellulose into glucose through the hydrolysis process. CMC is used as a substrate, it is hydrolyzed by endoglucanase, also known as CMCase [34]. Apart from endoglucanases, there are also exocellulases or cellobiohydrolases, and cellobiases or β-glucosidases. Exoglucanase are known to be active against microcrystalline substrate but inactive against CMC [35]. Bhadra et al. [35] reported several endophytic fungi capable of producing cellulase, *Pestalotiopsis* sp., *Acremonium* sp., *Microsphaeropsis* sp., *Sclerocystis* sp., *Nigrospora *sp., *Phomopsis* sp., *Keteleeria davidiana*, *Cephalosporium* sp., *Penicillium* sp., *Fusarium oxysporum*, *Aspergillus* sp., *P*.* chrysogenum*, and *Xylaria* sp.

Isolates Y2, Y4, and Y5 were found to qualitatively produce cyanide-degrading enzymes (Fig. 3). Glucosidase enzyme activity of endophytic fungal isolates obtained from cassava plants show in Figure 5. Among these, isolate Y2 exhibited the highest glucosidase enzyme activity (2.19 U/ml), followed by isolates Y4 (1.78 U/ml) and Y5 (1.96 U/ml). Cyanide is released by cyanogenic glycosides. Cyanogenic glycosides are secondary metabolites used by plants as a defense mechanism against various threats, such as bacteria, fungi, insects, and other predators. the cyanogenic glycosides are hydrolyzed by the β-glucosidase enzyme [36,37]. β-glucosidase enzyme plays a key role in the degradation of cellulose and other carbohydrates present in cell walls [38]. It does so by hydrolyzing the β-d-glycosidic bonds, thereby releasing glucose at the non-reducing ends of glucosides and oligosaccharides [39].

Amplification and comparison of fungal-specific ribosomal rRNA sequences are useful for morphological confirmation of identified fungi. Furthermore, molecular analysis aids in studying the phylogenetic and evolutionary relationships between different groups of endophytic fungi [40]. The 18S rRNA gene sequencing identified the isolate Y2 to belong to the *Phomopsis* sp. (Fig. 7). Amplification of 18S rRNA gene of endophytic fungal isolates shown in Figure 6. The sequencing results of the 570 bp amplicon showed a match with *Phomopsis* sp. The BLAST results for isolate Y2 (Table 1) showed that Y2 has homology or similarity to *Phomopsis* sp. 32PG/F of 99.82% at 98% query coverage. The sequence homology level is high with a score of ≥ 200. Isolate Y2 is registered in GenBank under the accession number PQ817715. *Phomopsis* is a genus of ascomycete fungi in the family *Valsaceae*. The genus *Phomopsis* has previously been found to be endophytic in plants. *Phomopsis* found in *Paris axialis* plants produces 4,5-dihydroxy-3-(2-hydroxyethyl)-1-methoxy-8-methoxycarbonylxanthone and 1,8-dihydroxy-4-(2-hydroxyethyl)-3-methoxy xanthone. While *Phomopsis* obtained from *Paris polyphylla* var. *yunnanensis* produces 1,5-dihydroxy-3-(2-oxopropyl)-6-methoxycarbonylxanthone and 1-hydroxy-3-(2-oxopropyl)-8-methoxycarbonyl xanthone. Jiang et al. [41] further explained that *Phomopsis* sp. produces compounds such as polyketides, alkaloids, peptides, terpenoids, and nucleosides, which possess antibacterial, anti-inflammatory, and cytotoxic properties.

**Table 1.** BLAST analysis results of Y2 endophyte fungi isolates found in GenBank.

No	Description	Max Score	Total Score	Query cover	*E* value	Identity	Accession
1	*Phomopsis *sp. 32PG/F	1,017	1,494	98%	0.0	99.82%	GU066621.1
2	*Phomopsis *sp. isolate HS-J-18	1,012	1,472	99%	0.0	98.63%	ON845723.1
3	*Diaporthe uekeri* isolate SLHX3	991	1,455	97%	0.0	99.28%	KY565424.1
4	*Diaporthe uekeri* isolat SLHX11	989	1,453	97%	0.0	98.28%	KY565425.1
5	*Diaporthe tectonendophytica *isolat MBEFPNJ05A	979	1,428	99%	0.0	97.42%	OQ123539.1
6	*Diaporthe longicolla *strain M12	976	1,417	99%	0.0	97.26%	PP421982.1
7	*Phomopsis longicolla *strain MP4PL11PS	948	1,386	98%	0.0	97.03%	HQ130441.1
8	*Phomopsis asparagi *isolat L5N87	948	1,371	97%	0.0	97.34%	MF185336.2
9	*Phomopsis asparagi *isolat L5N146	947	1,370	97%	0.0	97.18%	MF185354.2
10	*Diaporthe longicolla *strain DL17	943	1,381	97%	0.0	97.01%	MF125054.1

## Conclusion

Six fungal isolates isolated from cassava plants resulted in four isolates producing cellulose and three isolates producing cyanadase. Isolate Y2 is the isolate with the best activity for cellulase and cyanidase degrading enzymes, 2.99 U/ml and 2.19 U/ml, repectively. After molecular identification using 18S rRNA, isolate Y2 had 98.82% similarity to *Phomopsis* sp 32PG/F.

## List of abbreviations

CMC, carboxy methyl cellulose; CNP, cyanogenic potential; DM, dry matter; DNA, deoxyribonucleic acid; DNSA, 3,5 dinitrosalicylic acid; EDTA, ethylenediaminetetraacetic acid; EF, endophytic fungi; HCN, hydrocyanic acid; ITS, internal transcribed spacer; KCN, potassium cyanide; PDA, potato dextrose agar; pH, potential of hydrogen; rRNA, ribosomal ribonucleic acid; TBE, tris-borate-EDTA.
